# Hybrid Transcatheter and Endoscopic Retrieval of a Pulmonary Artery–Embolized ASD Occluder in Hemodynamic Compromise

**DOI:** 10.1016/j.jaccas.2026.107553

**Published:** 2026-03-25

**Authors:** Hristian Hinkov, Daniel Schwarzkopf, Dustin Greve, Chong Bin Lee, Christian Krall, Christoph Klein, Jörg Kempfert, Axel Unbehaun, Markus Kofler

**Affiliations:** a Department of Cardiothoracic and Vascular Surgery, Deutsches Herzzentrum der Charité (DHZC), Berlin, Germany; b Charité-Universitätsmedizin Berlin, Corporate Member of Freie Universität Berlin and Humboldt-Universität zu Berlin, Berlin, Germany; c Structural Heart Interventions Program (SHIP), Deutsches Herzzentrum der Charité (DHZC), Berlin, Germany; d DZHK (German Center for Cardiovascular Research), Partner Site Berlin, Berlin, Germany; e Department of Cardiology, Angiology and Intensive Care Medicine, Deutsches Herzzentrum der Charité (DHZC), Berlin, Germany; f Department of Cardiac Anaesthesiology and Intensive Care Medicine, Deutsches Herzzentrum der Charité (DHZC), Berlin, Germany

**Keywords:** Amplatzer occluder, ASD, device embolization, endcoscopic cardiac surgery, hybrid cardiac surgery, minimally invasive cardiac surgery, transcatheter retrieval

## Abstract

**Background:**

Embolization of atrial septal defect (ASD) occluders is uncommon but potentially life-threatening, often requiring urgent intervention. Transcatheter retrieval is typically attempted first, but success depends on achieving slenderizing of the device—which may be limited by anatomy or device position, frequently necessitating surgical removal.

**Case Summary:**

A 25-year-old woman presented with hemodynamic compromise after embolization of an ASD occluder into the main pulmonary artery. A tailored hybrid strategy was undertaken: controlled snare-guided mobilization of the device into the right ventricle created a surgically accessible position, enabling minimally invasive 3D-endoscopic extraction and definitive ASD repair, avoiding sternotomy and pulmonary arteriotomy.

**Discussion:**

This case represents the first reported hybrid combination of transcatheter approach with minimally invasive endoscopic cardiac surgery for pulmonary artery–embolized ASD occluder retrieval, highlighting the importance of interdisciplinary, cross-subspecialty procedural planning.

**Take-Home Message:**

Hybrid transcatheter-endoscopic approaches may be a feasible, least-invasive retrieval and repair strategy when standalone transcatheter retrieval is unsuccessful.

## Visual Summary

**Figure:**
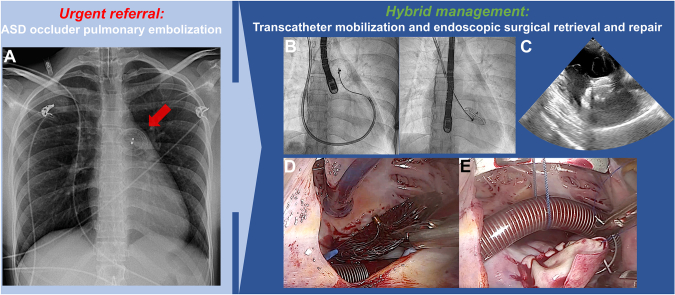

**Visual Summary:**
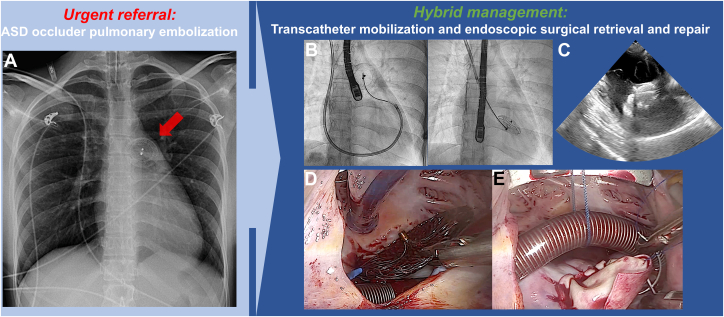
Central Illustration of Hybrid Transcatheter and Endoscopic Retrieval of a Pulmonary Artery–Embolized ASD Occluder in Hemodynamic Compromise (A) Chest radiograph showing the 26-mm Amplatzer atrial septal occluder projecting over the bifurcation of the main pulmonary artery (red arrow). (B) Transjugular snaring of the device using a steerable sheath and multipurpose catheter, followed by controlled mobilization into the right ventricle (RV). (C) Transesophageal echocardiography demonstrating the device within the RV at the level of the tricuspid subvalvular apparatus. (D) Endoscopic minimally invasive retrieval of the device from the RV through a right lateral microthoracotomy. (E) Definitive surgical closure of the atrial septal defect with a pericardial patch. ASD = atrial septal defect.

## History of Presentation

**Figure 1:**
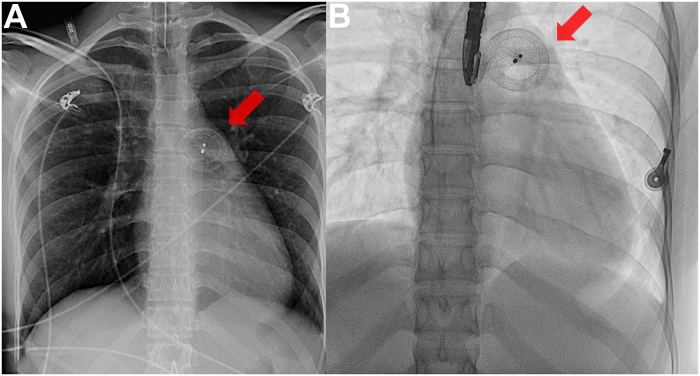
Chest Radiograph Prior Referral and Intraprocedural Baseline Fluoroscopy (A) Chest radiograph showing the 26-mm Amplatzer atrial septal occluder projecting over the bifurcation of the main pulmonary artery (red arrow). (B) Baseline intraprocedural fluoroscopy demonstrating the unchanged embolized position of the device (red arrow).

**Figure 2:**
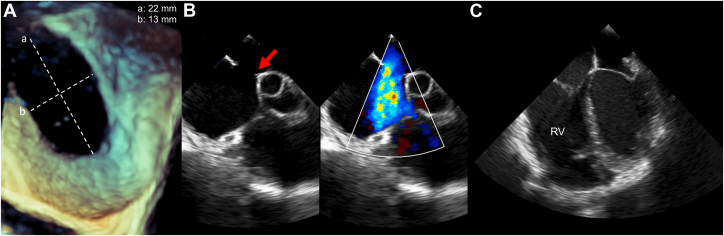
Echocardiographic Atrial Septal Defect Assessment Prior Index Intervention (A) Transesophageal echocardiographic (TEE) view demonstrating a 2.2 × 1.3-cm secundum-type atrial septal defect. (B) Two-dimensional and color Doppler imaging showing an insufficient aortic rim (white arrow) and a significant left-to-right shunt. (C) Four-chamber TEE view and short-axis parasternal transthoracic echocardiography view illustrating right ventricular (RV) enlargement.

A 25-year-old woman was urgently transferred to our center from a secondary-care hospital after elective percutaneous closure of an atrial septal defect (ASD) was complicated by embolization of the occluder into the main pulmonary artery (PA), causing hemodynamic compromise (Figure 1). The patient had a 2.2 × 1.3 cm secundum-type ASD, resulting in a left-right shunt with a pulmonary-to-systemic flow ratio of 2.4 and right ventricular (RV) dilation (Figure 2). RV systolic function was preserved. She was symptomatic for decreased exercise tolerance with increasingly recurrent exertional presyncopies and had no other past medical history. A percutaneous transfemoral attempt to retrieve the 26-mm Amplatzer Septal Occluder (Abbott Structural Heart), slenderizing it by a snare in a large-bore, transfemorally introduced 17-F sheath, proved unsuccessful at the external hospital. No further ASD repair attempts were undertaken. The patient was transferred intubated with an invasive arterial blood pressure of 90/50 mm Hg, supported by norepinephrine at 0.05 μg/kg/min, and a heart rate of 95 beats/min upon arrival.

**Take-Home Messages**

• A hybrid strategy combining a transcatheter approach with minimally invasive endoscopic surgery can further reduce operative invasiveness, highlighting the value of multidisciplinary heart teams with broad cross-subspecialty expertise.
• In ASD occluder pulmonary artery embolization, when in-sheath slenderizing is not feasible, controlled snare-guided mobilization into the right ventricle can position the device safely for surgery without causing tricuspid valve injury.
• Parking the device in the right ventricle enables single-stage, minimally invasive endoscopic extraction and definitive ASD patch repair through a right microthoracotomy, thereby avoiding both sternotomy and pulmonary arteriotomy.

## Past Medical History

The young patient had no prior medical history.

## Differential Diagnosis

The primary differential diagnosis was acute cardiac decompensation caused by a sudden increase in RV afterload due to central PA obstruction in the setting of an unrepaired high-flow left-to-right shunt. Additional considerations included acute RV failure from device-induced outflow tract obstruction, pericardial tamponade, air embolism, or sedation-related hemodynamic deterioration. However, imaging and clinical findings were most consistent with compromised pulmonary blood flow from device embolization.

## Investigations

As the patient had already undergone a complete diagnostic evaluation at the referring institution and arrived in an emergency setting with ongoing vasopressor support, no additional preprocedural investigations were performed. An interdisciplinary heart team—comprising cardiac anesthesia, an endoscopic/minimally invasive cardiac surgeon, an interventional cardiac surgeon, and an interventional cardiologist—discussed the management strategy at the bedside. In light of the patient's instability and the position of the embolized device, the team agreed on a hybrid approach aimed at achieving full or partial percutaneous retrieval of the occluder into an endoscopically/minimally invasively accessible position, permitting subsequent extraction through a right lateral microthoracotomy. This strategy was chosen to avoid median sternotomy in a young woman, preserving cosmesis and enabling minimally invasive surgical patch closure, whereas percutaneous reclosure did not seem feasible given the patient's anatomy. The patient was then transferred immediately to a hybrid operating theater.

## Management

With the patient under general anesthesia in the hybrid operating room, an 11-F sheath was placed in the right internal jugular vein. Through this access, a 145° pigtail catheter was placed in the right PA. Care was taken to avoid getting entangled within the subvalvular apparatus using transesophageal echocardiography (TEE) guidance. The pigtail catheter was exchanged for an Amplatz Super Stiff Guidewire (Boston Scientific) over which an 8.5-F steerable guiding sheath (Oscor) and a 7-F multipurpose catheter (Boston Scientific) were advanced, allowing the introduction of a 7-F 3D EnSnare system (Merit Medical). Slenderizing the occluder into the steerable sheath proved unfeasible. The embolized Amplatzer device was successfully grasped and withdrawn into the RV without causing pulmonary valve injury, as verified by real-time TEE. However, the occluder could not be advanced across the tricuspid valve without exerting significant traction or risking leaflet or chordal damage. Further retrograde mobilization was therefore deemed unsafe, especially as surgical repair of the ASD was considered.

Given the substantial risk of structural injury, the heart team proceeded with planned surgical rescue, leaving the occluder safely parked in the RV for endoscopic minimally invasive surgical retrieval. Cardiopulmonary bypass (CPB) was instituted following ultrasound-guided percutaneous cannulation of the common femoral artery and vein using a dual-stage RAP venous cannula (LivaNova). Surgical access was achieved through a 3-cm right lateral periareolar microthoracotomy in the fourth intercostal space, performed without rib spreading after deflation of both lungs. The surgical part of the procedure was performed fully endoscopically using a 3-dimensional scope (EinsteinVision 2.0 Aesculap system; B. Braun). The pericardium was opened anterior to the phrenic nerve, and myocardial protection was achieved with a single dose of 1,200 mL del Nido cardioplegia solution administered via the aortic root after transthoracic aortic cross-clamping. The superior and inferior vena cava were encircled, and a right atriotomy was performed to expose the tricuspid valve and RV. A sump catheter was introduced through the ASD for left ventricular venting.

**Figure 3:**
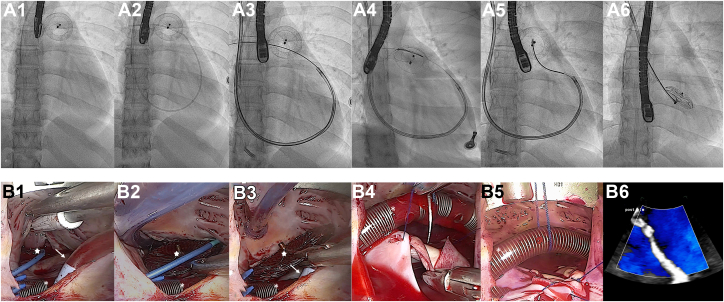
Hybrid Transcatheter-Minimally Invasive Endoscopic Retrieval of an Embolized Atrial Septal Defect Occluder (A1) Baseline fluoroscopy showing the 26-mm Amplatzer atrial septal defect (ASD) occluder (Abbott Structural Heart) projecting over the main pulmonary artery (PA). (A2) Engagement of the right PA with a 5-F angled pigtail catheter introduced through an 11-F right internal jugular sheath. (A3) Advancement of an Amplatz Super Stiff J-tip guidewire (Boston Scientific) through the pigtail catheter, enabling exchange to an 8.5-F steerable guiding sheath (Oscor). (A4) Introduction of a 7-F 3D EnSnare system (Merit Medical) via a 7-F multipurpose catheter through the steerable sheath. Initial snaring of one device pin was successful, but in-sheath slenderizing failed, and the occluder re-embolized to the PA bifurcation (not shown). (A5) Repeat snaring was achieved; however, coaxial alignment and slenderizing remained unsuccessful. (A6) The snared device was mobilized en bloc with the multipurpose catheter and steerable sheath and intentionally parked in the right ventricle. (B1) After establishing cardiopulmonary bypass via percutaneous femoral cannulation with a dual-stage venous cannula (white asterisk), minimally invasive endoscopic access was obtained through a right lateral microthoracotomy. Following cardioplegic arrest and right atriotomy, the parked occluder (white star) is visualized through the tricuspid valve. Left ventricular venting is performed via the ASD using a sump catheter (white arrow). (B2) Release of the EnSnare from the occluder pin (white star) and freeing of the device from the tricuspid subvalvular apparatus. (B3) Endoscopic extraction of the embolized device. (B4) Exposure of the ASD facilitated by anterior suspension of the venous cannula, followed by surgical patch repair. (B5) Endoscopic view after completion of the ASD patch repair. (B6) Transesophageal echocardiography confirming complete closure without residual shunting on color Doppler.

The RV-parked occluder was then released from the snare and extracted using forceps under direct visualization. Definitive repair of the ASD was accomplished with implantation of an autologous pericardial patch. The patient was weaned uneventfully from CPB, with an aortic cross-clamp time of 31 minutes and a total CPB time of 48 minutes. Total hybrid procedure duration was 202 minutes. Intraoperative TEE confirmed complete closure of the ASD patch and the absence of tricuspid valve injury. The procedural steps are illustrated in Figure 3 and Video 1. The postoperative course was uncomplicated, with a 1-day stay in the intensive care unit and a total hospital stay of 5 days (Video 2).

## Discussion

Embolization of ASD occluders is an uncommon complication, with reported incidences ranging from approximately 0.5% to 1.7%, depending on patient anatomy, device choice, and implantation technique.^1^ Despite the reliability of contemporary self-centering double-disc devices, migration may occur when anchoring support is inadequate, most often due to a deficient aortic septal rim, undersizing, or malpositioning.^2^ When embolization occurs, it is detected in most cases within the first 24 hours, and the device most commonly migrates into the main PA, as reported in up to 89% of cases. Device embolization can lead to significant hemodynamic compromise or vascular injury.^3^

Management strategies depend on the device's location and respective anatomical feasibility of transcatheter or surgical retrieval, as well as the clinical urgency determined by hemodynamic stability and potential downstream ischemia. Percutaneous retrieval is usually attempted first, but reported success rates vary widely—from 5% to 75%—reflecting heterogeneous anatomy, device position, and technique. Some series note that surgical retrieval is required in up to 77% of patients once dislocation has occurred. Consistent with these observations, several authors describe particularly pulmonary embolized ASD occluders as effectively irretrievable by endovascular means alone. In such cases, the standard surgical approach typically involves median sternotomy and pulmonary arteriotomy,^4^ which adds procedural morbidity and is generally undesirable.

Refinements in transcatheter retrieval have therefore been explored. Bench-test data highlight key determinants of successful extraction: sheath oversizing of at least 2-F beyond the original delivery system to allow controlled in-sheath slenderizing recapture, with the prerequisite of coaxial alignment of the device, which reduces the risk of frame or sheath-tip deformation during withdrawal,^5^ and the double-sling technique—sequentially using a snare and second snare-over-the-snare to improve grasping stability and coaxiality, but also slenderizing.^6^ Notably, in published series using this approach, PA embolization accounted for only a minority of cases,^7^ reflecting the difficulty of achieving coaxial alignment from a non-colinear venous trajectory to the PA. Hence, the additional challenge of pulling a nonslenderized device across both the pulmonary and tricuspid valves before attempting recapture in the right atrium or caval veins arises, where axial alignment is more readily attained.

Hybrid retrieval strategies for Amplatzer devices displaced into the aorta have been described, including transcatheter mobilization into the iliac or femoral vessels for retrieval through a laparotomy or retroperitoneal vascular cutdown, as well as catheter-based repositioning into the ascending aorta for subsequent transsternal surgical removal.^8^, ^9^, ^10^

To the best of our knowledge, this is the first case in which a transcatheter approach was combined with a minimally invasive, endoscopic right microthoracotomy to retrieve an occluder embolized into the PA, avoiding both pulmonary arteriotomy and sternotomy.

## Follow-Up

The patient was discharged home asymptomatic. Discharge echocardiography confirmed complete closure of the ASD with no residual shunt, normal right ventricular function, and intact tricuspid and pulmonary valves without regurgitation. A 6-month follow-up with reassessment of right ventricular size and pulmonary pressures is planned at the referring institution.

## Conclusions

This case demonstrates that combining transcatheter maneuvers with minimally invasive endoscopic cardiac surgery can provide an effective hybrid solution for managing PA embolized ASD occluders. A steerable sheath was used to optimize coaxial alignment for transcatheter retrieval after failure of a larger 17-F system at the referring institution. When adequate slenderizing could not be achieved, controlled snare-guided repositioning and parking of the device into the RV enabled single-stage endoscopic extraction and definitive ASD patch repair, thereby avoiding pulmonary arteriotomy and sternotomy. This hybrid strategy provides a feasible alternative in anatomically constrained situations where purely transcatheter retrieval is unlikely to succeed. It leverages the standard minimally invasive/endoscopic access used for ASD patch repair, particularly when a percutaneous redo ASD occlusion appears unpromising.

## Funding Support and Author Disclosures

Dr Unbehaun serves as a physician proctor for Edwards Lifesciences and Medtronic. Dr Kofler serves as a physician proctor for Edwards Lifesciences and EnableCV. All other authors have reported that they have no relationships relevant to the contents of this paper to disclose.

## Appendix

Video 1 Hybrid Transcatheter-Minimally Invasive Endoscopic Retrieval of an Embolized Atrial Septal Defect Occluder Hybrid procedural video summarizing the transcatheter and minimally invasive endoscopic surgical steps, including respective transesophageal echo findings.

Video 2 Case Video Summary Summary video illustrating the clinical presentation, hybrid procedural steps, and clinical course of the patient.
